# Antisense lncRNA *CHROMR* is linked to glioma patient survival

**DOI:** 10.3389/fmolb.2023.1101953

**Published:** 2023-03-06

**Authors:** Dovydas Širvinskas, Giedrius Steponaitis, Rytis Stakaitis, Arimantas Tamašauskas, Paulina Vaitkienė, Daina Skiriutė

**Affiliations:** ^1^ Laboratory of Molecular Neurobiology, Neuroscience Institute, Medical Academy, Lithuanian University of Health Sciences, Kaunas, Lithuania; ^2^ Laboratory of Molecular Neurooncology, Neuroscience Institute, Medical Academy, Lithuanian University of Health Sciences, Kaunas, Lithuania

**Keywords:** ncNATs, lncRNA, CHROMR, PRKRA, glioma, LGG, GBM, survival

## Abstract

**Background:** Natural non-coding antisense transcripts (ncNATs) are long non-coding RNAs (lncRNA) transcribed from the opposite strand of a separate protein coding or non-coding gene. As such, ncNATs can increase overlapping mRNA (and the coded protein) levels by stabilizing mRNA, absorbing inhibitory miRNAs and protecting the mRNA from degradation, or conversely decrease mRNA (or protein) levels by directing the mRNA towards degradation or inhibiting protein translation. Recently, growing numbers of ncNATs were shown to be dysregulated in cancerous cells, however, actual impact of ncNATs on cancer progression remains largely unknown. We therefore investigated gene expression levels of natural antisense lncRNA *CHROMR* (Cholesterol Induced Regulator of Metabolism RNA) and its sense protein coding gene *PRKRA* (Protein Activator of Interferon Induced Protein Kinase EIF2AK2) in gliomas. Next, we checked *CHROMR* effect on the survival of glioma patients.

**Methods:** We performed RNA-seq on post-surgical tumor samples from 26 glioma patients, and normal brain tissue. Gene expression in TPM values were extracted for *CHROMR* and *PRKRA* genes. These data were validated using the TCGA and GTEx gene expression databases.

**Results:** The gene expression level of ncNAT lncRNA *CHROMR* in glioma tissue was significantly higher compared to healthy brain tissue, while the expression of its sense counterpart protein coding *PRKRA* mRNA did not differ between glioma and healthy samples. Survival analysis showed lower survival rates in patients with low mRNA *PRKRA*/lncRNA *CHROMR* gene expression ratio compared to high ratio showing a link between lncRNA *CHROMR* and glioma patient survival prognosis.

**Conclusion:** Here we show that elevated levels of lncRNA *CHROMR* (i.e., low ratio of mRNA *PRKR*A/lncRNA *CHROMR*) is associated with poor prognosis for glioma patients*.*

## 1 Introduction

Natural antisense transcripts (NATs) are coding or non-coding RNA (ncRNA) sequences that are transcribed from the opposite strand of non-protein coding (nc) or protein coding (pc) genes and are complementary to or overlap with either protein-coding or non-coding transcript (Latgé et al., 2018; Santos et al., 2022). NATs can modulate the expression of their sense gene pairs (sense/antisense pairing) or of several related genes and are implicated in a broad variety of biological and pathological processes, including oncogenic progression (Guttman et al., 2009; Sun et al., 2017; Zhao et al., 2020; Krappinger et al., 2021). Most NATs are ncRNAs, among them long non-coding RNAs (lncRNAs), therefore most paired transcripts are composed of nc/nc or nc/pc pairs (Latgé et al., 2018). Regarding genomic position relative to its paired transcript, cis-NATs pairs are transcribed from the opposite strand of the same genomic locus and displays perfect RNA/RNA sequence complementarity with the opposite strand transcript (if there are no RNA modifications). Trans-NATs pairs are transcribed from different genomic loci with imperfect sequence complementarity (Latgé et al., 2018). Among cis-NATs, the estimated proportion of protein coding genes with ncNAT expression is thought to be up to 30%–50% in humans (Guttman et al., 2009; Latgé et al., 2018).

At the structural level ncNATs such as long non-coding RNAs may be multi-exonic, alternatively spliced, 5’capped, and 3’polyadenylated (Halley et al., 2013; Latgé et al., 2018). RNA polymerase II is responsible for the transcription of most lncRNAs, and its expression is controlled by promoters and enhancers, which can be induced by external stimuli (Guttman et al., 2009). NATs can drive important physiological events exerting its biological effects at small amounts (Yoshigai et al., 2014). It was shown that NAT transcription varies among cell and tissue types, and its expression is closely associated with paired sense gene (Lin et al., 2015; Zhao et al., 2020). Functionally antisense lncRNAs either promote or suppress cell proliferation, migration, and therapeutic resistance in tumors, and affect multiple stages of gene expression from epigenetic, transcriptional, to post-transcriptional, and translational modulations (Liu et al., 2021). Among the potential clinical applications of NATs, the non-coding/coding transcript pairs are of high interest for treatment (Latgé et al., 2018). However, the biological and clinical importance of NATs remains under investigation with many questions to be answered.

Gliomas account for up to 80% of malignant brain tumors, glioblastomas (GBM) being the most aggressive and malignant type (Rong et al., 2022). Despite progress in standard care of GBM patients, the outcome remains critically poor, ranging from 14.6 to 20.5 months, shortened to 8.5 months from diagnosis in elderly patients (Stupp et al., 2017). Due to the high mortality rates, caused by subpar treatment efficiency, and late detection of the tumor, the elucidation of molecular mechanisms of this disease is urgently needed.

To date, several studies have been performed to investigate the role of non-coding NATs in the context of glioma pathogenesis. Several lncRNAs, located on the antisense strand of a paired protein-coding gene (and often named after the protein coding gene, including an ‘–AS’ ending) have been linked to glioma: high levels of *PTB-AS* (Zhu et al., 2019), *ZEB1-AS1* (Meng et al., 2018), *HMMR-AS1* (Bao et al., 2021) promoted migration and proliferation in glioma, negatively impacting patient survival rates, while *ST7-AS1* (Sheng et al., 2021) was associated with prolonged patient survival acting as a tumor suppressor. It is thus readily apparent that lncRNAs acting as ncNATs are critical regulators of gene expression playing an important role in gliomas, impacting patient survival rates. Thus, further inquiry into known and novel ncNATs could provide new opportunities for glioma detection and treatment.

Long non-coding RNA *CHROMR* (Cholesterol Induced Regulator of Metabolism RNA) (a.k.a. *AC009948.5, CHROME* and *PRKRA-AS1*) is found to be involved in controlling cholesterol homeostasis in humans, by sponging inhibitory micro RNAs (miRNAs) miR-27b, miR-33a, miR-33b and miR-128 (Hennessy et al., 2019). In addition, recent data, has linked *CHROMR* with several types of cancer (Bai et al., 2022; Luo et al., 2022; Wang et al., 2022), antiviral response (van Solingen et al., 2022) and autoimmune disease (Teimuri et al., 2018). Based on the location of *CHROMR* in the Human genome with respect to the protein-coding gene, in GENECODE 7 annotation (Derrien et al., 2012) it is categorized as biotype “non-coding natural antisense transcript”, as the 3′ end of *CHROMR* overlaps with the 3′ end of its paired sense gene *PRKRA.* However, there is no published evidence of *CHROMR* antisense regulation of its paired coding gene *PRKRA* in gliomas yet.

*PRKRA* (Protein Activator of Interferon Induced Protein Kinase EIF2AK2) encodes the ‘Protein Activator of PKR’ (PACT) protein, which is the activator of *EIF2AK2* (‘RNA-activated protein kinase R’ (PKR), encoded by Eukaryotic Translation Initiation Factor 2 Alpha Kinase 2) gene. Under cellular stress, the PACT protein is phosphorylated, then binds to PKR, which leads to eIF2α (Eukaryotic Initiation Factor 2 alpha) phosphorylation, subsequently a halt to protein synthesis and leads to cell apoptosis (Patel et al., 2000). Conversely TRBP (TAR-RNA Binding Protein), encoded by the *TARBP2* (Trans-Activation-Responsive RNA-binding Protein) gene, binds to PKR and inhibits its’ ability to phosphorylate eIF2α (Daher et al., 2001). PACT also binds with TRBP and Dicer in the RNA-induced silencing complex (RISC), where it is important to produce small interfering RNA (siRNA) and process miRNA (Lee et al., 2006; Kok et al., 2007).

In this study, we investigated the expression profiling of the most poorly understood ncRNAs species—non-coding natural antisense transcripts (ncNATs) and identified significantly upregulated expression of antisense lncRNA *CHROMR* in glioma as compared to normal brain tissue using RNA-seq. We found that highly upregulated *CHROMR* in gliomas is associated with poor patient survival in TCGA samples. Further, *CHROMR* regulatory role on its paired protein coding sense gene *PRKRA* mRNA was checked in the same patient sample. *PRKRA* mRNA*/CHROMR* gene expression ratio was significantly lower in tumorous tissues than in normal brain samples and was associated with shorter patient survival.

## 2 Materials and methods

### 2.1 Patient samples

In total 26 post-surgical glioma samples: 9 grade-II diffuse astrocytomas, IDH mutant (here referred as Low-Grade Gliomas—LGG), and 17 grade-IV Glioblastomas, IDH wild-type (GBM) were collected between 2012 and 2021 at the Hospital Lithuanian University of Health Sciences “Kauno Klinikos”, Department of Neurosurgery. Samples were snap-frozen and stored in liquid nitrogen immediately after tumor resection. Tumor diagnosis was confirmed, and tumors were classified according to the WHO classification system (2016 fourth edition update 3) (Louis et al., 2016) by the Pathology Department at Lithuanian University of Health Sciences “Kauno Klinikos”. Written consent of participation in the study was obtained from all patients before tumor resection. Permission for scientific research on glioma patients’ tissue and clinical data was obtained from the Kaunas Regional Biomedical Research Ethics Committee—No, BE-2-26-2021.

### 2.2 RNA isolation

100 mg of snap-frozen tissue was used to isolate total RNA, utilizing TRIzol™ reagent (Invitrogen, cat. No 15596026). The RNA integrity was estimated by a native agarose gel (1.5%) electrophoresis, evaluating 28S and 18S RNA bands, which were visible around 5k and 2k nucleotide marker point (Invitrogen™, cat. No. AM7150). The approximate quantity of the extracted RNA was measured by the NanoDrop™ 2000 spectrophotometer (Thermo Scientific™, cat. No. ND-2000). Human normal brain tissue RNA (1 mg/mL) was obtained from Invitrogen (cat. No. AM7962).

### 2.3 Poly-A enrichment

On average, 84.6 µg of total RNA was enriched for poly-A tailed RNAs using Dynabeads™ mRNA DIRECT™ purification kit (Invitrogen™, cat. No. 61012). Next, the poly-A enriched RNA was precipitated overnight at −80°C in a precipitation buffer (final concentrations: 10% of 3 M sodium acetate (pH 5.52) (Sigma-Aldrich, cat. No. 32319-500G-R), 100 μg/mL of glycogen (Thermo Scientific™, cat. No. R0551), and 2,5 vol of pure ethanol. Finally, precipitated RNA was resuspended in 15 µL of RNase-free water and evaluated using the RNA 6000 Pico kit (Agilent, cat. No. 5067-1513) on 2100 Bioanalyzer instrument (Agilent, cat. No. G2939BA).

### 2.4 Direct RNA sequencing

Sequencing libraries were made from 600 ng of poly-A enriched RNA, applying Direct RNA sequencing kit (Oxford Nanopore Technologies (ONT), cat. No. SQK-RNA002) and following manufacturer’s protocol (ver. DRS_9080_v2_revO_14 August 2019). The only correction to this protocol was made by replacing the original Reverse Transcription Adapter (RTA) to a barcoded RTA, designed by Hyeshik Chang^1^. All samples were divided into groups of 4, except for two glioblastoma samples, which were grouped together. Samples in each group were individually barcoded during library preparation and sequenced together. Pooling of barcoded samples (50 ng each) was done after the reverse transcription reaction (after step 8 in the protocol). Next, purified libraries were quantified with Qubit™ dsDNA HS assay kit (Invitrogen™, cat. No. Q32851) on a Qubit™ four Fluorometer (Invitrogen™, cat. No. Q33238). Finally, 200 ng of libraries were sequenced for approximately 48 h on a R9.4.1 flow cell (ONT, cat. No. FLO-MIN106D) using MinION or MinION Mk1C sequencers.

### 2.5 Processing of sequencing data, NATs selection criteria

Fast5 files were base-called with high accuracy mode on using ONT’s Guppy software (Version v5.0.11) applying a configuration file for dRNA-seq (“rna_r9.4.1_70bps_hac.cfg”). Base-called fast5 files were processed with a Poreplex software (Version 0.5) to extract fastq files for each barcoded sample. Next, the fastq files were aligned to the full Ensembl’s reference transcriptome (GRCh38. p13; Version 105; cDNA + ncRNA) (Cunningham et al., 2022) using Minimap2 software (Settings*: ax map-ont -p 0 -N 10*; Version 2.17-r941) (Li, 2018). Aligned SAM files were sorted and indexed with Samtools (Version 1.15.1) (Danecek et al., 2021) and quantified using NanoCount (Settings: *-min_alignment_length 50 --min_query_fraction_aligned 0.5 --sec_scoring_value alignment_score --sec_scoring_threshold 0.95 --convergence_target 0.005 --max_em_rounds 100*; Version v1.0.0. post6) (Gleeson et al., 2022). A list of all currently identified NATs was downloaded from the Guigó Lab (Derrien et al., 2012) and merged with NanoCount’s output. Of all overlapping intronic antisense lncRNA/mRNA pairs, the *CHROMR* and *PRKRA* pair had the highest (18/26%–69%) number of samples with both transcripts detected in our data. Our analysis included only these samples (GBM *n* = 11; LGG *n* = 7). All the computational work was done on a GenomeDK server, using CentosOS (Release 7.9.2009) and Miniconda virtual environment (Anaconda Software Distribution, V ersion 22.9.0).

### 2.6 External datasets used for analysis

Publicly available LGG and GBM data was obtained from The Cancer Genome Atlas (TCGA) dbGaP study accession phs000178^2^. Publicly available healthy brain tissue data was obtained from the Genotype-Tissue Expression portal (GTEx) dbGaP accession phs000424. v9^3^. Publicly available proteomic data of healthy brain and glioma was obtained from The Human Protein Atlas version 21.1, Ensembl version 103.38^4^, (Uhlén et al., 2015).

### 2.7 Statistical analyses

Statistical calculations and graphical preparations were done on the GraphPad Prism (Version 9.4.1 (681)) software. The non-parametric ANOVA Kruskal-Wallis test with Dunn’s multiple comparisons test was used for ratio comparisons. Survival analysis performed using Kaplan-Meier survival curve comparison analysis.

## 3 Results

### 3.1 Antisense lncRNA *CHROMR* and sense protein coding *PRKRA* are differentially expressed in brain gliomas

**FIGURE 1:**
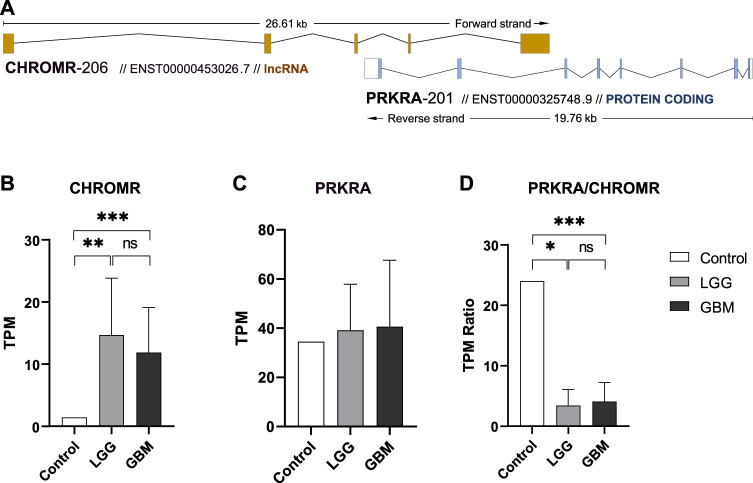
ncNAT CHROMR and its overlapped protein coding gene PRKRA expression in glioma and normal brain tissue. **(A)** Schematic view of lncRNA CHROMR-206 (ENST00000453026.7, + strand) and mRNA PRKRA-201 (ENST00000325748.9,—strand) transcript genomic overlap as annotated in Ensembl genome browser. Exons are depicted as colored boxes. **(B–D)**. Our in-house RNAseq data of healthy brain (control), LGG and GBM samples for lncRNA CHROMR **(B)**, mRNA PRKRA **(C)** genes and mRNA PRKRA/lncRNA CHROMR expression ratio **(D)**. N (Control, LGG, GBM) = 1; 7; 11, respectively, one-sample *t*-test. *—*p* < 0.05; **—*p* < 0.01, ***—*p* < 0.001.

Natural antisense lncRNA *CHROMR* (ENST00000453026.7) according to NATs annotation in GENECODE v7 database (Derrien et al., 2012) belongs to *cis* overlapping antisense lncRNA transcribed at the same genomic loci as its paired protein coding gene. According to the orientation with respect to the sense protein coding gene *PRKRA* (ENST00000325748.9), lncRNA *CHROMR* could be classified as tail-to-tail overlapping intronic NAT (Figure 1A). We therefore decided to check the expression levels of both genes as well as their possible link with the survival of glioma patients.

We extracted lncRNA *CHROMR* and mRNA *PRKRA* gene expression data from our in-house glioma patient RNA-seq dataset. Here we found that LGG and GBM samples had more than 3.0 log2-fold (3.35 and 3.04, respectively) significantly higher expression of lncRNA *CHROMR* than healthy brain (Figure 1B), while expression of protein coding *PRKRA* mRNA did not seem to differ between healthy brain and glioma samples (Figure 1C). This reduced the mRNA *PRKRA/*lncRNA *CHROMR* ratio in LGG and GBM samples by a log2-fold change of 2.81 and 2.57, respectively as compared to normal brain tissue (Figure 1D), which suggests a high expression of lncRNA *CHROMR* is characteristic of brain gliomas. Interestingly, protein-level data obtained from the Human Protein Atlas database suggests levels of *PRKRA* encoded protein PACT are lower in gliomas as compared to normal brain tissue (Supplementary Figure S1), but the low number of samples and qualitative nature of the data raise doubts about the validity of such results.

**FIGURE 2:**
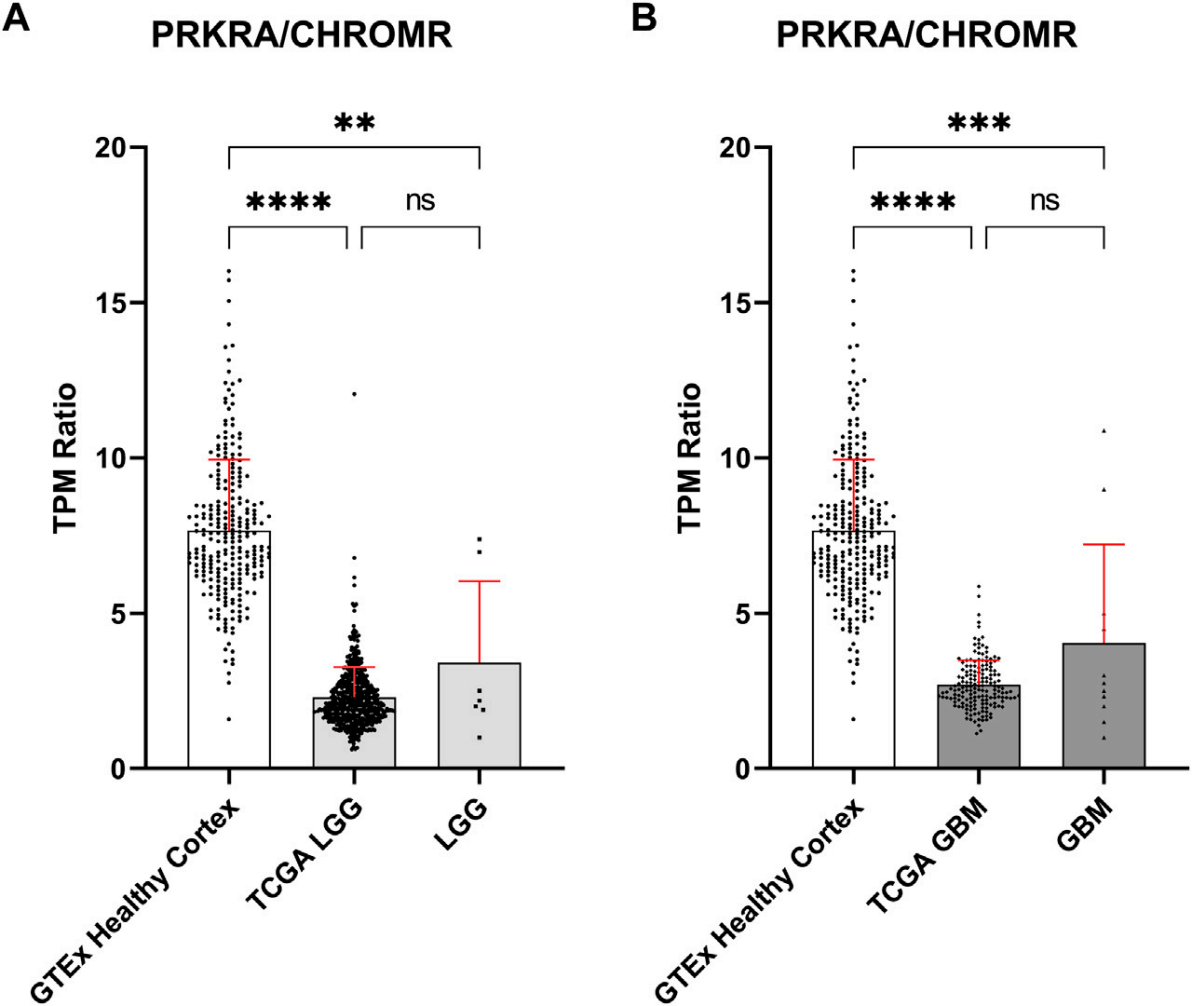
Comparison of mRNA PRKRA/lncRNA CHROMR gene expression ratio in LGG and GBM. **(A,B)** Comparing mRNA PRKRA/lncRNA CHROMR expression ratio of GTEx database Healthy Cortex samples with LGG **(A)** and GBM samples **(B)**. N (GTEx Healthy Cortex, TCGA LGG, TCGA GBM) = 255; 532; 168; respectively. LGG and GBM here denotes our in-house RNA-seq data. Data is represented as mean with standard deviation. Kruskal-Walli’s test with Dunn’s multiple comparison test. ns—*p* > 0.05; ** - *p* < 0.01; *** - *p* < 0.001, **** - *p* < 0.0001.

To validate these findings, we obtained publicly available data through the GTEx and TCGA databases for normal brain and glioma samples respectively, for comparison with our in-house generated data. Owing to possible TPM (Transcripts Per Million) differences due to sequencing depth variation between our and database samples, comparisons were focused on the mRNA *PRKRA/*lncRNA *CHROMR* ratio (Figures 2A,B). This should theoretically eliminate any effect of technical variation on compared results.

The *PRKRA/CHROMR* ratio was consistent between glioma samples: we did not find a statistically significant difference for *PRKRA*/*CHROMR* ratio between TCGA patient and our patient samples. There was a statistically significant difference for *PRKRA/CHROMR* ratio when comparing pathological (both LGG and GBM) and healthy (brain cortex) brain tissue expression, confirming our previous observation from our in-house RNA-seq data. (Figures 2A,B). This confirms that a lower mRNA *PRKRA/*lncRNA *CHROMR* ratio (and thus a high expression of lncRNA *CHROMR*) is a feature of brain glioma.

### 3.2 *PRKRA/CHROMR* gene expression ratio has prognostic value in brain glioma

**FIGURE 3:**
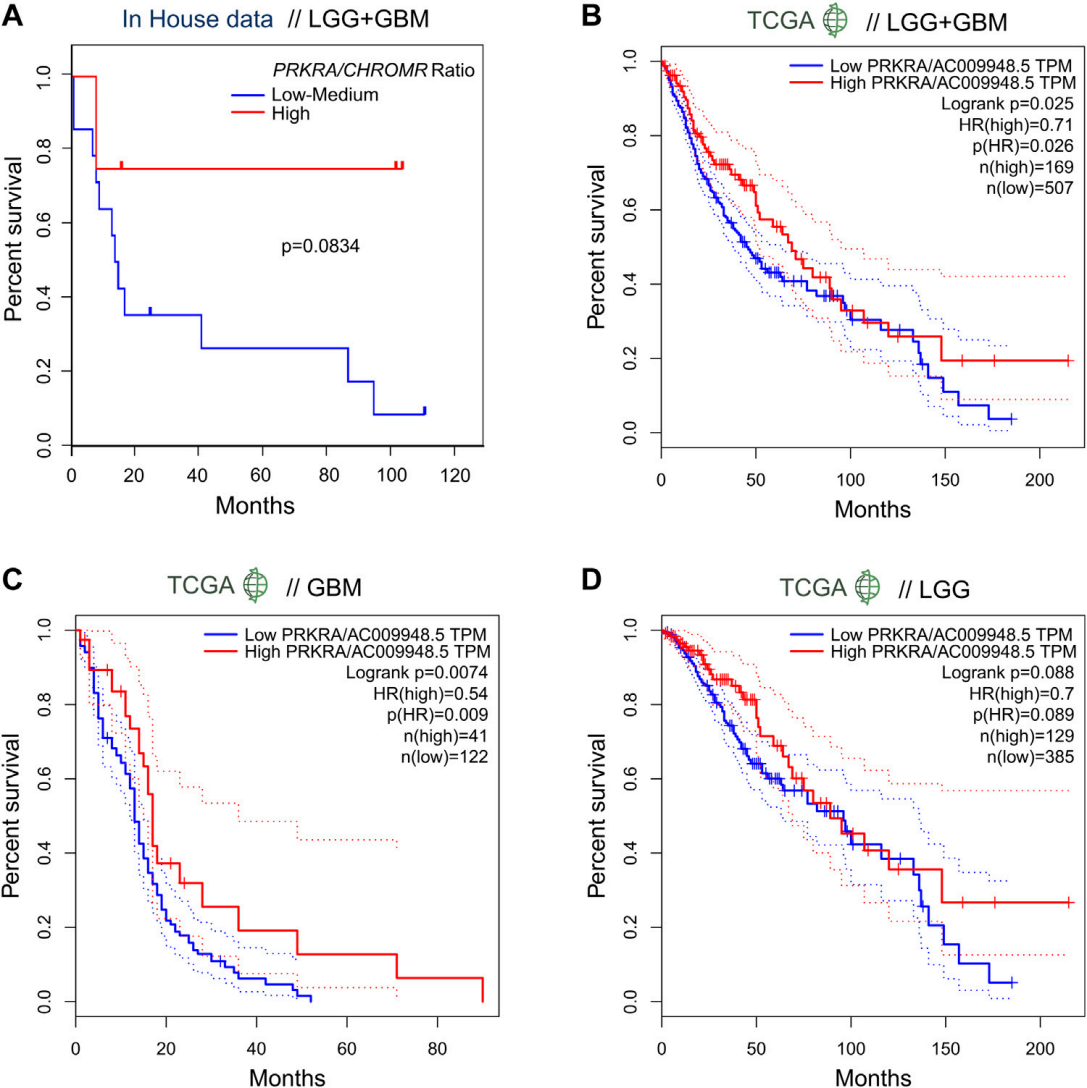
Expression ratio of mRNA PRKRA/lncRNA CHROMR affects survival ratio of LGG and GBM patients. **(A)** Comparison of survival probability for pooled LGG and GBM patients with High mRNA PRKRA/lncRNA CHROMR expression Ratio (above third quartile) and Low-medium expression Ratio (under third quartile). **(B–D)** GEPIA tool generated patient survival curves comparing mRNA PRKRA/lncRNA CHROMR High and Low gene expression (in TPM) ratio groups in pooled LGG and GBM datasets **(B)** and separately **(C,D)**, dotted lines indicating 95% Confidence Interval of the survival curve. N (TCGA LGG, TCGA GBM) = 514; 163; respectively. Mantel-Cox (Log-rank) test. Statistically significant - *p* < 0.05. HR denotes hazard ratio.

In order to evaluate the effect of the mRNA *PRKRA/*lncRNA *CHROMR* on glioma patient outcome, we performed survival analysis. Here we show that patients (LGG + GBM) with a high *PRKRA/CHROMR* ratio (above the third quartile) had a higher survival rate than those below the third quartile (Hazard Ratio = 0.2), though our data did not reach statistical significance (Log-rank test, *p* = 0.0834) (Figure 3A). Comparing high (above median) and low (below median) expression of lncRNA *CHROMR* (Supplementary Figure S2A) and mRNA *PRKRA* (Supplementary Figure S2B) individually, we could not find a statistically significant hazard ratio either, possibly due to the relatively low number of patients in our cohort.

To overcome this, we checked the survival rates of glioma patients from the TCGA database using the GEPIA online tool^5^ (Tang et al., 2017). When provided with analogous cutoff inputs (above the third quartile *versus* below the third quartile), we found a statistically significant Hazard Ratio of 0.71 for mRNA *PRKRA/*lncRNA *CHROMR* (Figure 3B) and 1.4 for lncRNA *CHROMR* (Supplementary Figure S2C), but not *PRKRA* (Supplementary Figure S2F) in LGG + GBM samples.

Interestingly, we were able to observe a difference between LGG and GBM patient survival according to our genes of interest. Unlike the combined LGG + GBM dataset, the effect of *PRKRA*/*CHROMR* ratio did not reach statistical significance in TCGA LGG patient outcomes (*p* = 0.088) (Figure 3D). However, the TCGA LGG patients had a significantly higher hazard ratio for high lncRNA *CHROMR* expression by itself (HR = 1.7, *p* = 0.0033) (Supplementary Figure S2D). No effect of *PRKRA* (Supplementary Figure S2G) could be observed on the survival of LGG patients.

When analyzing TCGA GBM samples, however, we saw that only the *PRKRA*/*CHROMR* ratio (Figure 3C), but not *CHROMR* (Supplementary Figure S2E) or *PRKRA* (Supplementary Figure S2H) separately, had prognostic value: GBM patients who had a high *PRKRA*/*CHROMR* ratio had a significantly lower hazard ratio (HR = 0.54, *p* = 0.0074). These results highlight that high lncRNA *CHROMR* is detrimental to glioma patient survival. However, it has to be emphasized that for GBM, both *PRKRA* and *CHROMR* have to be taken into account, as *CHROMR* alone is not informative in patient survival prognosis.

## 4 Discussion

Natural antisense transcripts (NATs) are coding or non-coding RNA sequences transcribed from the opposite direction from the same genomic locus as overlapped genes and seem to play important roles in pathological processes (Latgé et al., 2018; Santos et al., 2022) through various molecular mechanisms (Zhao et al., 2020; Krappinger et al., 2021; Molias et al., 2021). Only in recent years due to evolvement of high-throughput strand-specific sequencing technologies lowly expressed NATs (to which antisense long non-coding RNAs belong) attracted attention as candidate biomarkers and therapeutic targets in cancer. Here we focus on the interesting role of natural antisense lncRNA *CHROMR* in glioma.

Some NATs are able to bind histones or recruit histone-modifying proteins to their genomic locus, exerting either a negative or positive epigenetic regulatory effect on neighboring genes (Magistri et al., 2012). There is also evidence of NATs exerting transcriptional interference, whereby transcription of the NAT obstructs transcription of the overlapped gene (Stojic et al., 2016). Other NATs act outside the nucleus, interacting with overlapped gene mRNA, which can stabilize the mRNA (Zhu et al., 2019) or facilitate alternative splicing (Yin et al., 2017). Additionally, NATs can capture, and ‘sponge’ miRNA intended to target the sense mRNA (Zeng et al., 2019). Therefore, it is evident that NATs have multiple avenues of effect and are no doubt important in many if not most processes in the cell.

The fact that *PRKRA*/*CHROMR* ratio, but not the genes separately, had an effect on survival of glioblastoma patients, suggests that *CHROMR* may indeed have some NAT-associated interaction with the overlapped *PRKRA* gene in glioblastoma, but not low-grade glioma. Since glioblastoma is the most aggressive and malignant type of brain tumor (Rong et al., 2022), associated with high mortality rates and poor treatment efficiency (Stupp et al., 2017), such findings could prove useful in elucidating the molecular mechanisms and/or improving available treatments for glioblastomas.

Though it could be expected that lncRNA overlapping sense mRNA would modulate the levels of overlapped gene mRNA, such an effect could not be observed regarding lncRNA *CHROMR* and protein coding *PRKRA*. Indeed, it has been shown that *CHROMR* shows a weak interaction with the *PRKRA* genomic locus and that *CHROMR* expression does not impact the expression of the *PRKRA* gene (van Solingen et al., 2022) at least in the context of macrophages. However, lncRNA are known for their ability to modulate gene activity without impacting levels of mRNA, so it is not yet conclusive that *CHROMR* does not interact with *PRKRA*. Interestingly, *CHROMR* has been shown to have histone binding affinity in macrophages (van Solingen et al., 2022), which could still be an avenue for *PRKRA* regulation, without binding to *PRKRA* mRNA. It is also known that *CHROMR* can act as a sponge for various miRNAs (Hennessy et al., 2019; Wang et al., 2022), but it would be difficult to attribute miRNA sponging to observed effects on glioma. For example, miR-27b, which *CHROMR* seems to be able to sponge (Hennessy et al., 2019) has both been linked with glioma progression (Chen et al., 2011; Liu et al., 2015; Miao et al., 2020) and glioma repression (Chang et al., 2020; ZHAN et al., 2021). Therefore, alternate means of *PRKRA*-*CHROMR* interaction are possible, but further experiments would need to be done to elucidate the mechanism.

*CHROMR* has also been implicated in elevated risk for stomach adenocarcinoma (Luo et al., 2022), lung adenocarcinoma (Bai et al., 2022), increased resistance to chemotherapy in Lymphoma (Wang et al., 2022), but also in antiviral response to Influenza and COVID-19 infections (van Solingen et al., 2022) and autoimmune disease, such as multiple sclerosis (Teimuri et al., 2018). *CHROMR* expression is different when comparing lymphoid cancer (high) with myeloid cancer (low) cell lines (Yang et al., 2022). Our results suggest that increased *CHROMR* expression could be linked to development of malignancy and poorer prognosis for brain glioma patients.

Protein coding *PRKRA* is important for normal brain function. Mutations of the *PRKRA* gene are responsible for the DYT-PRKRA (DYT16) dystonia (Camargos et al., 2008; Burnett et al., 2020; Vaughn et al., 2022), characterized by death of neurons in specific brain regions, causing involuntary movements. This effect comes from aberrant and increased stress response (Vaughn et al., 2022). Perhaps there is aberrant *PRKRA* protein function in glioma cells, resulting in a reduced ability to respond to pro-apoptotic signals, that could be caused by the observed high *CHROMR* expression in gliomas. This theory would, however, require further experiments to prove.

*PRKRA* has been implicated in conferring chemoresistance to mucinous ovarian cancer cells (Hisamatsu et al., 2019) and in antiviral response (Vaughn et al., 2022). It is still to be determined if *PRKRA* acts in a similar fashion in glioma as it does the mucinous ovarian cancer cells, as gliomas are notoriously resistant to chemotherapeutic agents. It has been shown, however, that endoplasmic reticulum stress makes glioma cells more susceptible to chemotherapeutic agents through PERK signaling (Xipell et al., 2016), which like *PRKRA*, acts *via* phosphorylation of eIF2α. It could mean *PRKRA* dependent chemoresistance works through a different pathway, but whether *PRKRA* even has a role in glioma chemoresistance is still to be demonstrated. It is also not known if and how *CHROMR* may impact *PRKRA* dependent chemoresistance.

Glioma cells are able to evade the anti-tumor immune response due to the cancer’s immunosuppressive repertoire (Chen et al., 2021). Since *PRKRA* is known to play a role in antiviral response (Vaughn et al., 2022), perhaps our observations of high levels of *CHROMR* in glioma could play a role in inhibiting *PRKRA* related antiviral (which is related to anti-tumor) response. This would, however, require functional experiments to prove.

Both protein coding *PRKRA* and lncRNA *CHROMR* seem to have diverse functions in different tissues and different diseases, therefore need to be investigated at a tissue and disease specific context. Here we report a link between glioma patient survival and the lncRNA *CHROMR* gene, which could be linked with the overlapped *PRKRA* gene in glioblastomas. However, further experiments will have to be performed in order to elucidate the mechanism of action and other players involved in the *CHROMR*-Glioma link.

## Data Availability

The dataset presented in the study are deposited in the NCBI Gene Expression Omnibus repository (https://www.ncbi.nlm.nih.gov/geo/), accession number GSE219250.

## Footnotes

1
https://github.com/hyeshik/poreplex.

2
https://www.cancer.gov/tcga.

3
https://gtexportal.org/home/datasets.

4
https://www.proteinatlas.org/about/download.

5
http://gepia.cancer-pku.cn/.
