# Supplementary material for: Antisense lncRNA CHROMR is linked to glioma patient survival
Source: Front Mol Biosci. 2023 Mar 6;10:1101953. doi: 10.3389/fmolb.2023.1101953 (PMC10025505; doi:10.3389/fmolb.2023.1101953)
Supplement: Supplementary file 1 [file Image2.pdf]

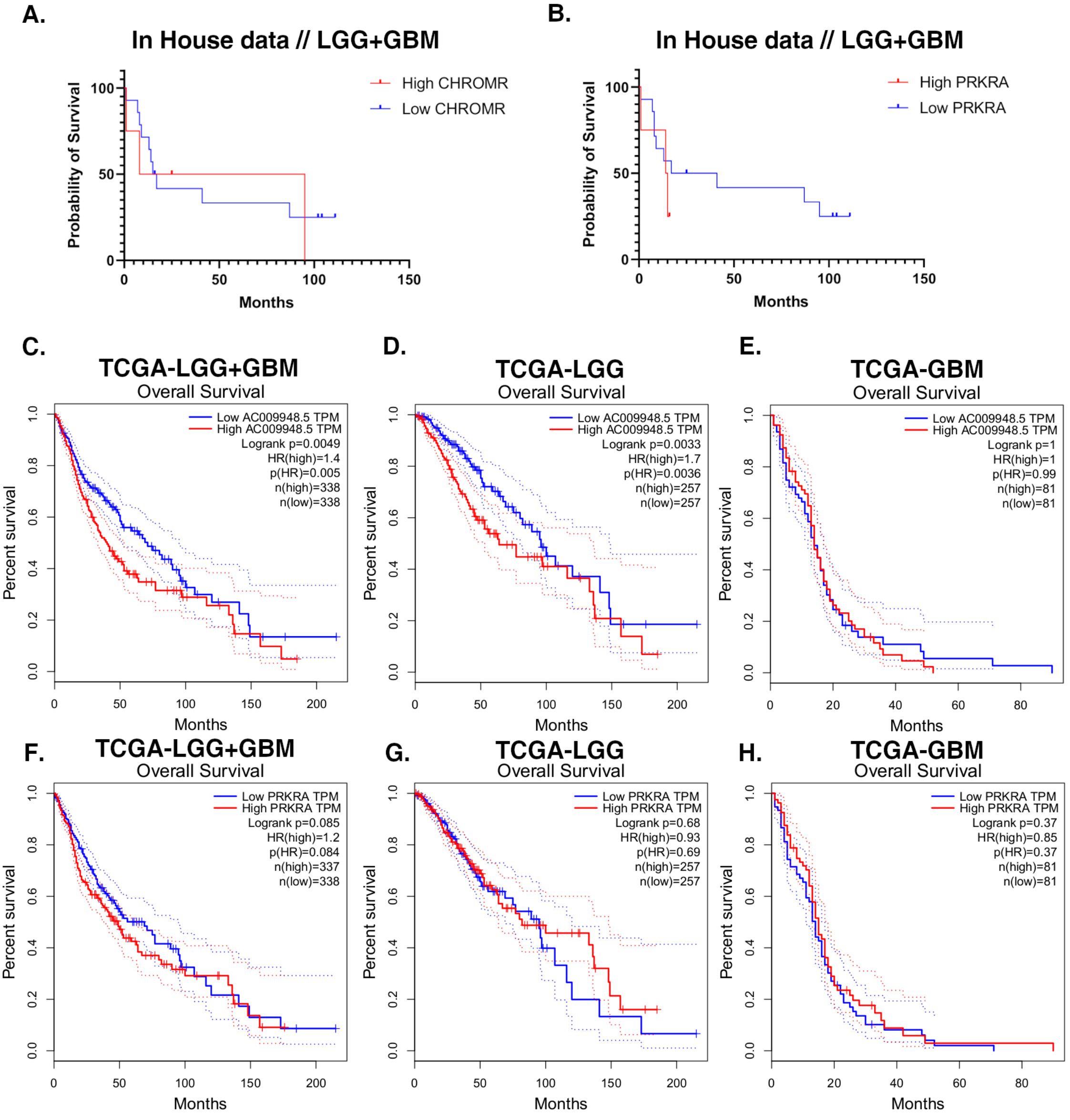

Figure S2: Survival of patients according to *PRKRA* and *CHROMR*. A-B Survival curves for LGG+GBM patients comparing High (above 3rd quartile) and Low (under 3rd quartile) expression of lncRNA *CHROMR* (A) and mRNA *PRKRA* (B). C-E Survival curve for TCGA database LGG+GBM (C), LGG (D) and GBM (E) patients, comparing expression of *CHROMR*. F-H. Survival curves for TCGA database LGG+GBM (F), LGG (G) and GBM (H) patients, comparing expression of *PRKRA*.
