# Supplementary material for: Antisense lncRNA CHROMR is linked to glioma patient survival
Source: Front Mol Biosci. 2023 Mar 6;10:1101953. doi: 10.3389/fmolb.2023.1101953 (PMC10025505; doi:10.3389/fmolb.2023.1101953)
Supplement: Supplementary file 2 [file Image1.pdf]

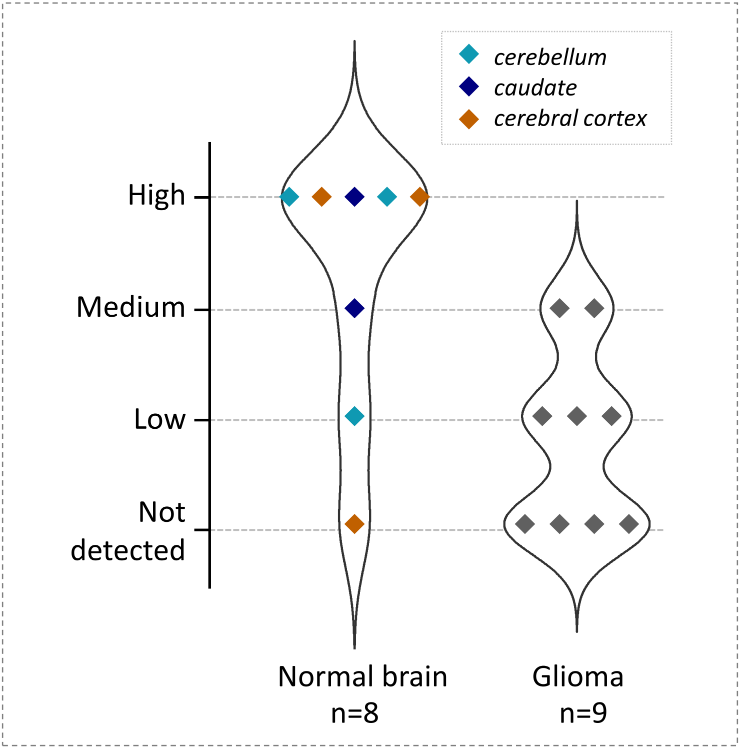

FIGURE S1

PACT protein levels in healthy brain and glioma, according to the Human Protein Atlas database. Protein expression score is based on immunohistochemical data manually scored regarding staining intensity: negative, weak, moderate, strong in the figure respectively corresponds as Not detected, Low, Medium and High.
